# Genetic modification of the shikimate pathway to reduce lignin content in switchgrass (*Panicum virgatum* L.) significantly impacts plant microbiomes

**DOI:** 10.1128/spectrum.01546-24

**Published:** 2024-11-26

**Authors:** Shuang Liu, Ming-Yi Chou, Gian Maria Niccolò Benucci, Aymerick Eudes, Gregory Bonito

**Affiliations:** 1 Department of Plant, Soil, and Microbial Sciences, Michigan State University, East Lansing, Michigan, USA; 2 DOE Great Lakes Bioenergy Research Center, Michigan State University, East Lansing, Michigan, USA; 3 Department of Plant Biology, Rutgers University, New Brunswick, New Jersey, USA; 4 DOE Joint BioEnergy Institute, Emeryville, California, USA; 5 Environmental Genomics and Systems Biology Division, Lawrence Berkeley National Laboratory, Berkeley, California, USA; USDA-ARS-NPRL, Dawson, Georgia, USA

**Keywords:** fungal and bacterial communities, bioenergy crops, genetic engineering, mycorrhizal fungi, actinobacteria

## Abstract

Switchgrass (*Panicum virgatum* L.) is considered a sustainable biofuel feedstock, given its fast-impact growth, low input requirements, and high biomass yields. Improvements in bioenergy conversion efficiency of switchgrass could be made by reducing its lignin content. Engineered switchgrass that expresses a bacterial 3-dehydroshikimate dehydratase (QsuB) has reduced lignin content and improved biomass saccharification due to the rerouting of the shikimate pathway towards the simple aromatic protocatechuate at the expense of lignin biosynthesis. However, the impacts of this QsuB trait on switchgrass microbiome structure and function remain unclear. To address this, wild-type and QsuB-engineered switchgrass were grown in switchgrass field soils, and samples were collected from inflorescences, leaves, roots, rhizospheres, and bulk soils for microbiome analysis. We investigated how QsuB expression influenced switchgrass-associated fungal and bacterial communities using high-throughput Illumina MiSeq amplicon sequencing of ITS and 16S rDNA. Compared to wild-type, QsuB-engineered switchgrass hosted different microbial communities in roots, rhizosphere, and leaves. Specifically, QsuB-engineered plants had a lower relative abundance of arbuscular mycorrhizal fungi (AMF). Additionally, QsuB-engineered plants had fewer *Actinobacteriota* in root and rhizosphere samples. These findings may indicate that changes in the plant metabolism impact both AMF and *Actinobacteriota* similarly or potential interactions between AMF and the bacterial community. This study enhances understanding of plant-microbiome interactions by providing baseline microbial data for developing beneficial bioengineering strategies and by assessing nontarget impacts of engineered plant traits on the plant microbiome.

**IMPORTANCE:**

Bioenergy crops provide an important strategy for mitigating climate change. Reducing the lignin in bioenergy crops could improve fermentable sugar yields for more efficient conversion into bioenergy and bioproducts. In this study, we assessed how switchgrass engineered for low lignin impacted aboveground and belowground switchgrass microbiome. Our results show unexpected reductions in mycorrhizas and actinobacteria in belowground tissues, raising questions on the resilience and function of genetically engineered plants in agricultural systems.

## INTRODUCTION

The biofuel industry has developed significantly over the past two decades given the impending need to replace fossil fuels and mitigate climate change (1, 2). Switchgrass (*Panicum virgatum* L.) is a perennial grass with C4 photosynthesis, an adaptation that anatomically separates the assimilation and reduction of CO_2_, thereby reducing photorespiration. Switchgrass is also a flagship sustainable biofuel feedstock species in North America given its wide native range, fast growth, high cellulose content, and relatively low requirements for water, nutrients, and pesticides (3–5). Lignocellulosic material is the cheapest feedstock to produce biofuels (6), and nearly 80% of switchgrass dry-weight biomass is composed of cellulose, hemicellulose, and lignin (7).

Lignin is a major plant cell wall component and in grasses is composed of large branched and oxygenated polyaromatic compounds consisting of monomer units of coniferyl, sinapyl, and *p*-coumaryl alcohols (6, 8). Since lignin contributes to biomass recalcitrance to deconstruction, reducing lignin content in feedstocks facilitates cellulose and hemicellulose hydrolysis, thus, increasing fermentable sugar yields from biomass and improving its conversion efficiency to bioenergy and advanced bioproducts (4, 9, 10).

Several genetic engineering techniques have been used to reduce lignin content in plants (11). These include the silencing of genes encoding lignin biosynthetic enzymes such as 4-coumarate: CoA ligase (10) and caffeate *O*-methyltransferase (12). Another promising strategy for reducing lignin in bioenergy crops involves the expression of bacterial 3-dehydroshikimate dehydratase (QsuB), which reduces the pool of precursors necessary for lignification (13). In the shikimate pathway, 3-dehydroshikimate is the precursor of shikimate and phenylalanine, which are key metabolites involved in lignin biosynthesis (14). QsuB converts 3-dehydroshikimate into protocatechuate and thereby limits lignin biosynthesis. Such genetic modifications have been shown to improve the saccharification of biomass compared to wild-type plants (13, 15). For example, the expression of QsuB in switchgrass resulted in a 12%–21% reduction in lignin content and 5%–30% increase in saccharification efficiency, as well as greater bioaccumulation of protocatechuate (16).

Plant-associated microbiomes are composed of populations of diverse bacteria and fungi that colonize internal and external plant tissues and may include beneficial, commensal, and pathogenic organisms. Microbiomes have been shown to be important for maintaining plant health and can be leveraged to increase biomass yield (17, 18), enhance plant nutrient availability (19), improve drought tolerance (20), and further provide other ecosystem services related to soil structure, water retention, and carbon storage (21, 22). For example, switchgrass plants inoculated with *Serendipita vermifera* (originally *Sebacina*) produced as much as 75% and 113% more shoot biomass at 2-month and 3.5-month harvests, respectively (17).

Plant genotype has been shown to be a factor involved in structuring plant microbiome, as was found for bacterial communities in the switchgrass rhizosphere, as well as aboveground and belowground fungal and bacterial microbiomes of switchgrass (23–25). Similar results were found for switchgrass phyllosphere microbiomes in the field (26). Changes in microbial community between highly productive and less productive switchgrass cultivars can be linked to the greater and lower microbial nitrogenase activity, respectively, which suggested a possible linkage between microbiomes and cultivar yields (27, 28).

Genetic engineering can improve plant biomass yield and chemical properties, but it may also have unexpected impacts on plant-microbe interactions. For example, the silencing of cinnamoyl-CoA reductase gene reduced lignin in poplar trees but also significantly changed the bacterial community in roots, stems, and leaves (29). Similarly, poplar trees downregulated in genes encoding for the lignin biosynthetic enzymes caffeoyl-CoA O-methyltransferase, caffeic acid O-methyltransferase, cinnamoyl-CoA reductase, and cinnamyl alcohol dehydrogenase all displayed a lower mycorrhizal colonization *in vitro* (30). DeBruyn et al. (31) reported that lower lignin lines of COMT (caffeic acid O-methyltransferase)-downregulated switchgrass plants had no effects on bacterial diversity, richness, or community composition of soil samples, but they did not investigate the fungal community and other plant compartments. Thus, although growing engineered switchgrass with reduced lignin could have obvious industrial advantages regarding deconstruction and conversion processes, with no phenotypic differences noted, the impact of the engineered trait on the structure and functioning of the plant microbiome needs to be evaluated.

In this study, we assessed the impact of QsuB-engineered switchgrass plants on the microbiome across plant compartments. We accomplished this by characterizing both fungal and bacterial communities, within bulk soil, rhizosphere, root, leaf, and inflorescence of QsuB and wild-type Cave-in-Rock switchgrass. We hypothesized that QsuB-engineered traits would alter the structure of fungal and bacterial microbiomes by reducing species richness and evenness, particularly in belowground samples that support high amounts of microbial diversity.

## MATERIALS AND METHODS

### Plant growth and transplant

The transgenic switchgrass line *pZmCesa10: QsuB-5* and parental wild-type (cultivar Alamo-A4) used in this study have been described previously (16). Three transgenic and three wild-type plants reared from tissue culture were raised in axenic conditions for 3 months and were then planted in sterile potting mix (Sure Mix, Michigan Grower Products Inc., Galesburg, MI, U.S.) and grown vegetatively (16 hr light and 8 hr dark at 23°C) for 6 months to establish sufficient biomass to allow each plant to be split into three genetically identical individuals. Deionized water was applied every other day, and 1:10 Hoagland fertilizer was applied every other week to prevent nutrient deficiency under potting condition. Splitting was done by excising each plant at the crown into three at approximately equal crown size with a sterilized scissor. The senesced aboveground tissues and old structural roots were trimmed off to only retain green aboveground tissue and minimum non-lignified young roots.

After splitting, switchgrass plants were planted into new pots with sand blended in with field soil, to provide a diverse microbiome inoculum. Field soil was collected from the top 20 cm of a switchgrass field in the long-term ecological research station for bioenergy cropping systems in Hickory Corner, MI, in August 2021 and was sieved through a 1-cm hardware cloth to homogenize and remove root fragments and organic debris before mixing with sterile sand. Homogenized sieved field soil was then mixed with double-autoclaved play sand 50/50 (vol/vol) to provide proper drainage of water in the pots. For microbiome analyses, nine biological replicates were used for both the wild-type and QsuB genotypes. Split plants were raised under the same conditions as described and used prior to splitting. After 3 months, the final microbiome sampling was conducted, and the experiment was terminated.

### Sample collection and processing

Sampling of above and belowground switchgrass-associated microbiomes was carried out at two separate occasions: after splitting prior to planting in field soil (as pre-transplant sampling status) and 3 months after splitting and planting in the field soil (as post-transplant sampling status). Samples were collected from two soil niches (i.e., bulk, rhizosphere) and four plant niches (root endosphere, leaf, inflorescence, senesced leaves). Bulk soil from triplicate plant splits was collected with a sterile spatula avoiding root zones. Rhizosphere soil was sampled from each replicate by collecting three young lateral roots up to 3 cm in length with root hairs included from each plant. Roots were vigorously agitated by hand to detach the loosely attached soil prior to washing roots. The roots were then collected in 2-mL Eppendorf tubes, filling 1/3 of the volume, and vortexed in ddH_2_O containing 0.05% Tween 20 for 20 min to dislodge the tightly attached soil. These root washes were kept as rhizosphere soil samples, which contained both rhizosphere and rhizoplane communities. Washed roots were then surface sterilized in 6% hydrogen peroxide solution for 30 s, rinsed twice with sterile ddH_2_O, and kept as root endophytic samples. Expanded young healthy leaves of each plant were sampled at splitting. Other aboveground tissues including inflorescence and senesced leaves were also sampled from each plant at the end of the experiment by using sterile scissors. All aboveground tissues were sampled at 5 cm below the tips for approximately 1 cm from three randomly picked tissue objects.

### DNA extraction and Illumina MiSeq sequencing

Samples were flash frozen in liquid nitrogen within 1 hr after collecting. Samples were then freeze-dried with a SpeedVac (Thermo Fisher, Waltham, MA, U.S.), placed in 2-mL centrifuge tubes together with three metal beads in each tube, and ground to a powder with a TissueLyser II (Qiagen, Hilden, Germany) at maximum speed for 40 s. Microbial DNA was extracted from soil samples with a MagAttract PowerSoil DNA kit (Qiagen, Hilden, Germany) and from plant samples with E.Z.N.A. Plant DNA kit (Omega Bio-Tek, Norcross, GA, U.S.). Libraries were prepared as previously described, including black samples as negative controls (24) with some modifications. Briefly, extracted DNA was amplified with primer sets 515 f and 806 r for bacterial communities targeting the 16S rDNA V4 region and primers 5.8 f and ITS4r for fungal communities targeting ITS2 rDNA (32, 33). Following the initial amplification, amplicons were PCR-ligated onto Illumina sequencing adapters and customized barcodes and normalized with a Norgen DNA purification kit (Norgen Biotek Corp., Thorold, ON, Canada). Pooled barcoded amplicons were then purified and concentrated with Amicon centrifugal units (Sigma-Aldrich, St. Louis, MO, U.S.) and further purified with a HighPrep PCR Clean-up System (MAGBIO Genomics, Gaithersburg, MD, U.S.). Sequencing was conducted at the Michigan State University RTSF Genomic Cores (East Lansing, MI, U.S.) with a v3 kit on an Illumina MiSeq sequencer. The raw sequences were demultiplexed with default setting in bcl2fastq, filtered, and clustered into amplicon sequence variants (ASVs) using DADA2 (34) in R 4.0.2. ASV taxonomic annotations were generated using CONSTAX2 v2.0.18 (35) with SILVA v138 (36) for the 16S and UNITE 9.0 (37) for the ITS regions, respectively. Raw 16S and ITS sequences data were deposited in NCBI under BioProject ID PRJNA1002602 and PRJNA1002603, respectively.

### Statistical analysis and data visualization

Microbial 16S and ITS rRNA amplicon sequence variant (ASV) tables, taxonomy tables, and metadata were imported into the R software for statistical computing and graphics. We removed ITS sequences with BLAST identity and coverage of ≤60% to the UNITE fungal database v 9.0 (38). Pre-transplant leaf samples and post-transplant senescence leaf samples were dominated by plant organelles (e.g., mitochondria, chloroplast) with a very low number of fungal and bacterial sequences; therefore, we removed these samples from our analysis (Fig. S1 and S2). Mitochondria and chloroplast sequences were also removed from the overall 16S data set. Sequence distributions allowed for the detection and removal of outlier samples, one post-transplant non-QsuB leaf sample and one post-transplant non-QsuB root sample, having low fungal read counts (Fig. S3 and S4). Samples with low number of reads (i.e., distribution outliers) were removed by adopting rarefaction cutoffs of 2,948 and 12,126 sequence reads per sample for fungi and bacteria, respectively. Rarefaction curves were calculated in the *vegan* package (39) and plotted in the *ggplot2* package (40). Rarefaction curves showed that most samples recover the whole diversity present in each sample, and rarefaction only marginally affected the total number of ASV detected across the entire data sets (Fig. S5).

Rarefied ASV richness and Shannon diversity index were calculated in *vegan*. Beta-diversity Bray-Curtis distance matrices were assessed to illustrate the community structures between samples and sample groups. We used nonmetric multidimensional scaling (NMDS) and principal coordinate analysis (PCoA) ordinations to visualize beta-diversity. Permutational multivariate analysis of variance (PERMANOVA) was performed to test the statistical differences of beta-diversity between sample groups. We tested the interaction between Niche and Treatment, Status and Treatment, and Status and Niche while controlling for Status, Niche, and Treatment, respectively. Since PERMANOVA (“adons2”, vegan R package) does not allow specifying random effects, we took advantage of the sequential nature of the function in calculating the sums of squares and specified the fix/random factor as first term in the model. To assess differences in dispersion between groups (i.e., multivariate heteroscedasticity) that can contribute to the group difference detected with adonis2, a multivariate dispersion analysis was used as implemented in the R function “betadisper”. To compare alpha diversity measures between groups (i.e., ASV richness and Shannon index), we used a nonparametric Wilcoxon signed-rank test with *P* values corrected for multiple comparisons using the Benjamin-Hochberg method. Stacked bar charts were generated to show the relative abundance of lineage-level bacteria and fungi in sample groups. To identify differentially abundant ASV across sample groups, we used a pairwise Wilcoxon test and DESeq2 in the *stats* (41) and *DESeq2* (42) R packages, respectively. All analysis and figures were generated in R (41), and the R code to reproduce the analysis is available here: https://github.com/Gian77/Scientific-Papers-R-Code/.

## RESULTS

### Data summary and overview

In total, we obtained 17,811,594 ITS and 30,086,564 16S raw sequence reads, respectively, from the 198 sample libraries. After removing nontarget ASVs, including non-fungal eukaryotes, chloroplasts, and mitochondria , a total of 13,403,151 ITS and 24,577,163 16S reads remained, respectively. These accounted for 8,089 and 33,853 ASVs for the ITS (fungal) and 16S (bacterial) communities, respectively, distributed across 144 total samples. On average, each sample had 93,077.44 (± 41,537.25 standard deviation) ITS sequence reads and 170,674.7 (± 110,850 standard deviation) 16S sequence reads. No sequences remained in negative control samples.

The three experimental variables in our design were (1) status (pre-transplant and post-transplant to field soil), (2) niche (bulk soils, rhizosphere soils, roots, leaves, and inflorescences), and (3) genotype (QsuB and non-QsuB wild-type). In the nonmetric multidimensional scaling analyses of fungal and bacterial data sets, samples from the same niche clustered together, especially in bacterial communities, when plotting on two dimensions (Fig. S6). Bulk soil and rhizosphere fungal and bacterial communities clustered with each other but apart from root and aboveground communities. Bacterial communities of belowground samples (roots, rhizosphere, and bulk soils) were distinct from those of aboveground samples (leaf and inflorescence) prominently. Pre- and post-transplant samples were also clearly separated in ordination space (Fig. S6).

Sampling status and sampling niches had obvious influences on both fungal and bacterial communities. Therefore, to investigate the influence of the genotype on microbial communities, we split our data sets by sampling niches and status in the following analysis.

### QsuB leaf and pre-transplant root samples had higher bacterial richness and diversity

**Fig 1:**
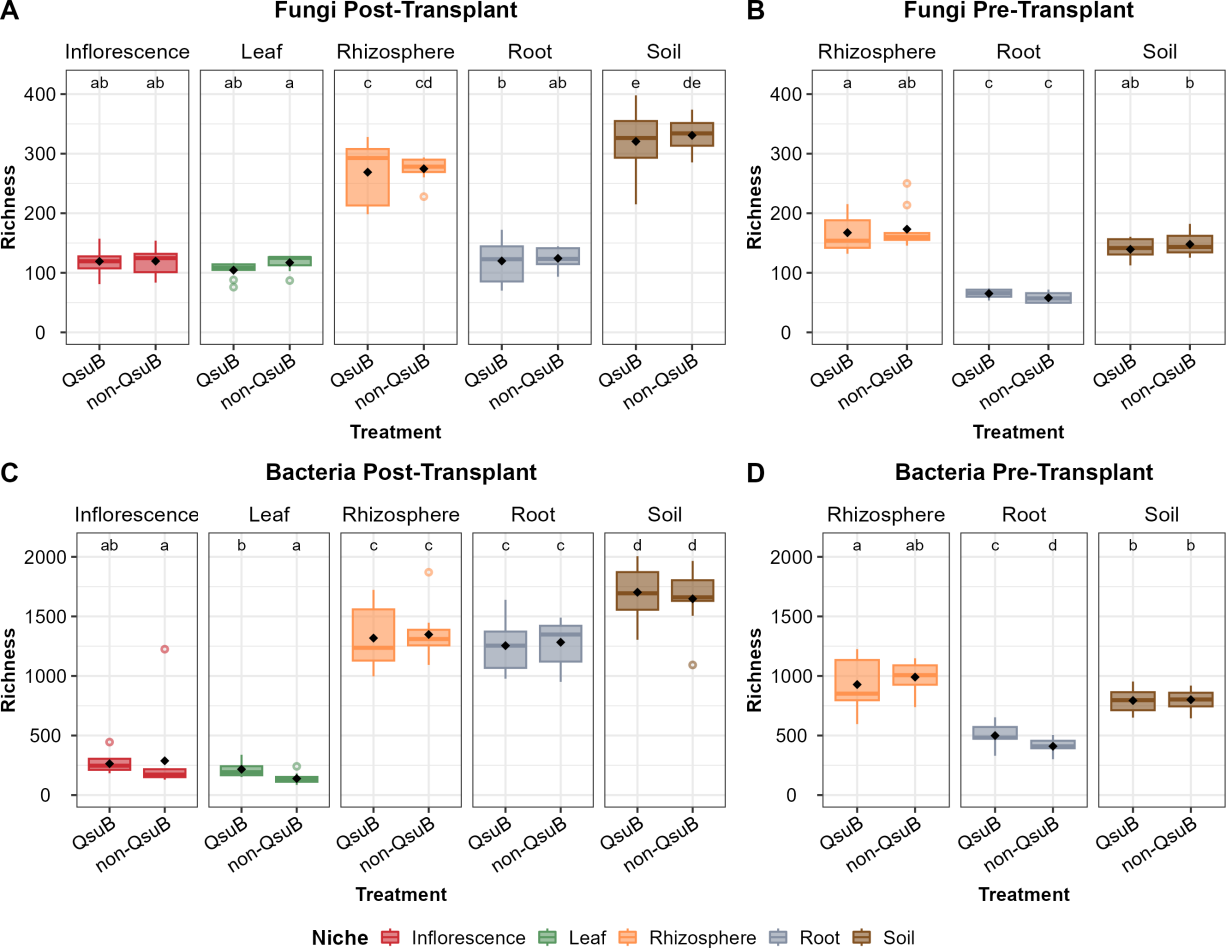
Boxplot of observed ASV richness for post-transplant fungi (**A**), pre-transplant fungi (**B**), post-transplant bacteria (**C**), and pre-transplant bacteria (**D**) grouped by sampling niches (inflorescence, leaf, rhizosphere, root, and soil). Letters represent pairwise Wilcoxon tests among groups (*P* ≤ 0.05 after Bonferroni adjustment).

In general, soil and rhizosphere samples had significantly higher richness than inflorescence and leaf samples (Wilcoxon test, *P < 0.05*) (Fig. 1). The QsuB genotype had no significant influence on the fungal richness in any plant niches of pre-transplant or post-transplant samples (Fig. 1A and B). However, QsuB plants had significantly greater bacterial richness in post-transplant leaf and pre-transplant root samples, but not in post-transplant root samples (Fig. 1C and D).

**Fig 2:**
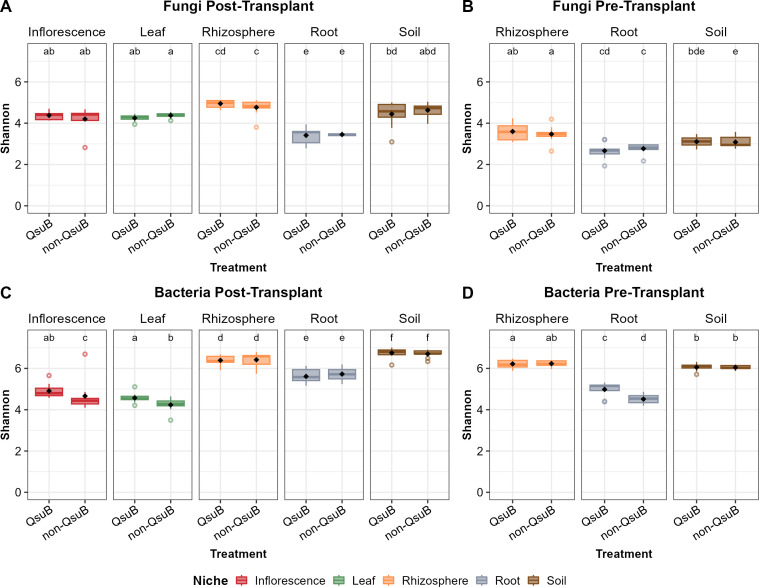
Boxplot of Shannon indices for post-transplant fungi (**A**), pre-transplant fungi (**B**), post-transplant bacteria (**C**), and pre-transplant bacteria (**D**) grouped by sampling niches (inflorescence, leaf, rhizosphere, root, and soil). Letters represent pairwise Wilcoxon tests among groups (*P* ≤ 0.05 after Bonferroni adjustment).

Fungal communities in root samples had significantly lower Shannon diversity compared to soil, rhizosphere, and aboveground tissues. The QsuB genotype had no significant influence on the fungal Shannon diversity indices across sampling niches for both pre-transplant and post-transplant samples (Fig. 2A and B). For bacterial communities, soil samples (soil and rhizosphere) had significantly greater diversity than plant samples (root, inflorescence, and leaf) (Fig. 2). The QsuB plants had significantly greater bacterial Shannon indices in post-transplant inflorescence, leaf, and pre-transplant root samples, but not in post-transplant root samples (Fig. 2C and D).

Additionally, it is worth noting that, for the same sampling niches, post-transplant samples always had greater bacterial and fungal richness and Shannon indices than those of corresponding pre-transplant samples (Fig. 1 and 2).

### QsuB significantly influenced root and leaf fungal community beta-diversity

**Fig 3:**
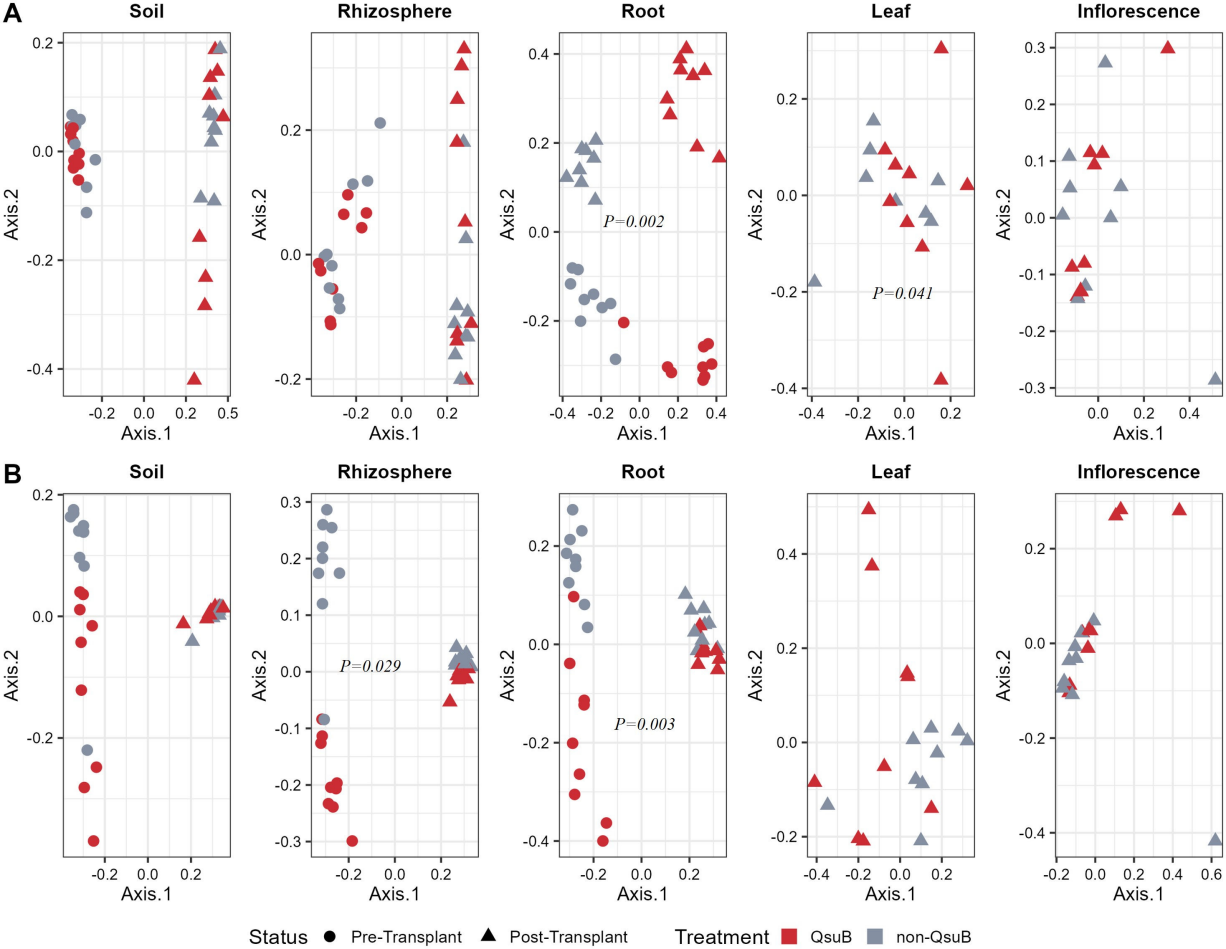
Principal coordinate analysis ordinations of fungal (**A**) and bacterial (**B**) communities sampled from soil, rhizosphere, root, leaf, and inflorescence niches at pre-transplant and post-transplant. Permutational multivariate analysis of variance was performed to test the statistical differences of beta-diversity. Less than 0.05 of *P values* on the graph indicated the significant influence of QsuB traits on communities between sample groups.

We used principal coordinate analysis ordinations to improve the visualization of beta-diversity results (Fig. 3A) and statistically examined the treatment effects on the beta-diversity. The QsuB traits significantly influenced the fungal community structures in the root (*P = 0.002*) and post-transplant leaf (*P = 0.041*) samples according to PERMANOVA (Table S1). In root samples, genotype, status, and the interaction between them were all significant factors of the fungal community structures. The QsuB genotype explained the most variance with the highest R^2^ of 23.09% (*P = 0.002*), followed by status (R^2^ = 17.68%, *P = 0.002*) and the interaction (R^2^ = 6.47%, *P = 0.003*) (Table S1). However, we also detected differences in multivariate variances between the groups we analyzed with PERMANOVA, i.e., the fungal community in root samples (Table S2).

### QsuB significantly influenced root and rhizosphere bacterial community beta-diversity

The root bacterial community of QsuB genotype and wild-type switchgrass separated from each other on two-dimension PCoA (Fig. 3B) and the visual observation was supported by the PERMANOVA. QsuB genotype significantly influenced the bacterial community structures in root (*P* = 0.003) and rhizosphere (*P* = 0.029) samples (Table S1). In both root and rhizosphere samples, genotype, status, and the interaction between them were all significant factors of the bacterial community structures. In root samples, the status explained the most variance with R^2^ of 20.56% (*P* = 0.003), followed by QsuB genotype (R^2^ = 5.67%, *P* = 0.003) and their interaction (R^2^ = 4.35%, *P* = 0.019) (Table S1). In rhizosphere samples, the status also explained the most variance with R^2^ of 21.31% (*P = 0.003*), followed by QsuB genotype (R^2^ = 4.15%, *P = 0.029*) and their interaction (R^2^ = 3.83%, *P = 0.029*) (Table S1). However, we did not detect differences in multivariate variances between the groups we analyzed with PERMANOVA in bacterial community (Table S2).

For both root and rhizosphere samples, the influence from the QsuB genotype was only manifested in pre-transplant samples in the PCoA plots, not in post-transplant samples (Fig. 3B). To focus on the impact of the QsuB genotype and eliminate interference from different status, we assessed beta-diversity on the post-transplant root and rhizosphere samples. This approach revealed that the QsuB genotype had significant influence on the bacterial community of post-transplant root (*P* = 0.001) and rhizosphere (*P* = 0.003) samples (Fig. S7; Table S1).

### Fungal composition

Overall, sixteen fungal lineages were detected in our sample. *Ascomycota*, *Glomeromycotina*, and *Basidiomycota* are predominant and have average relative abundance of 80.63%, 8.57%, and 7.97%, respectively (Fig. S8). Most *Glomeromycotina* were detected in root samples, and the majority of *Basidiomycota* were detected in post-transplant bulk soil and rhizosphere samples (Fig. S8). The above beta-diversity analyses showed that the QsuB genotype significantly influenced the fungal community of root and leaf samples. Nearly 94.00% of leaf fungi were *Ascomycota* (Fig. S8).

The significant effects of QsuB genotype on root fungal communities were likely explained by relatively more *Ascomycota* and relatively fewer *Glomeromycotina* in QsuB plants (Fig. S8). In post-transplant root samples, QsuB plants had 62.52% *Ascomycota* and 32.86% *Glomeromycotina*, while non-QsuB wild-type had 50.74% *Ascomycota* and 47.76% *Glomeromycotina*. Statistical analysis on lineage-level relative abundance data showed that only *Ascomycota* (*P* < 0.001) were significantly influenced by QsuB genotype in post-transplant root samples. However, in pre-transplant root samples, both *Ascomycota* (*P* = 0.009) and *Glomeromycotina* (*P* = 0.033) were significantly influenced by QsuB genotype. In pre-transplant root samples, QsuB plants had 78.35% *Ascomycota* and 18.57% *Glomeromycotina*, while non-QsuB wild-type had 67.63% *Ascomycota* and 27.71% *Glomeromycotina* (Fig. S8).

**Fig 4:**
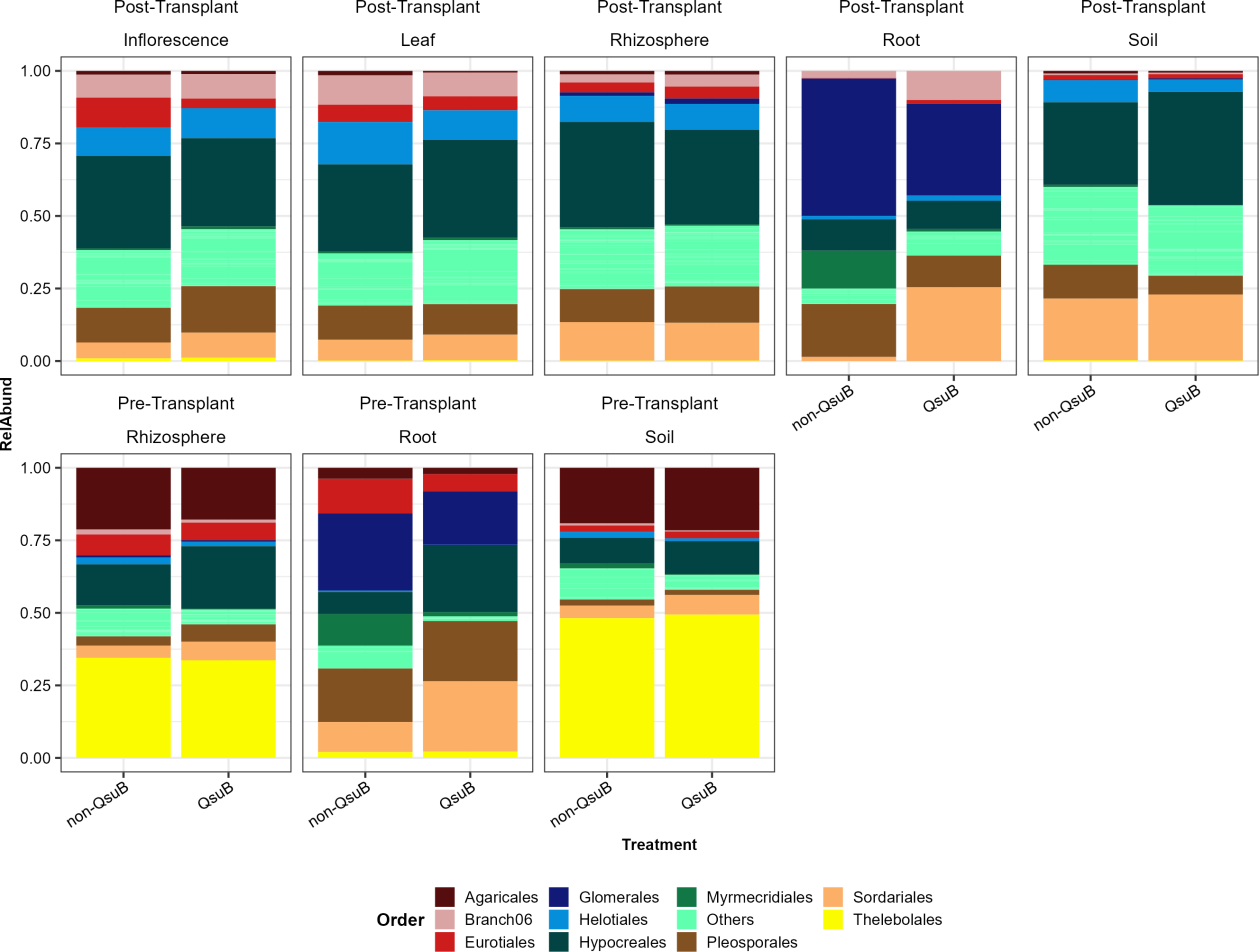
Order-level fungal taxonomic distribution of samples from inflorescence, leaf, rhizosphere, root, and bulk soil niches of post-transplant and pre-transplant.

To further investigate the QsuB genotype effects, we visualized the relative abundance of fungi at order level. We present the top 10 orders (by relative abundance), with the remaining orders detected represented as “Others” (Fig. 4). In pre-transplant root samples, three of top 10 orders were significantly influenced by QsuB genotype, and they are all from class *Sordariomycetes*. QsuB plants had significantly more *Hypocreales* (*P* = 0.021) and *Sordariales* (*P* = 0.010), but less *Myrmecridiales* (*P* = 0.005). Similarly, much more *Sordariales* and less *Myrmecridiales* in QsuB plants were also observed in post-transplant root samples. In pre-transplant samples, the relative abundance of *Glomerales* decreased from 27% in non-QsuB to 18% in QsuB, while in post-transplant samples, it decreased from 47% to 32% (Fig. 4).

### Bacterial composition

**Fig 5:**
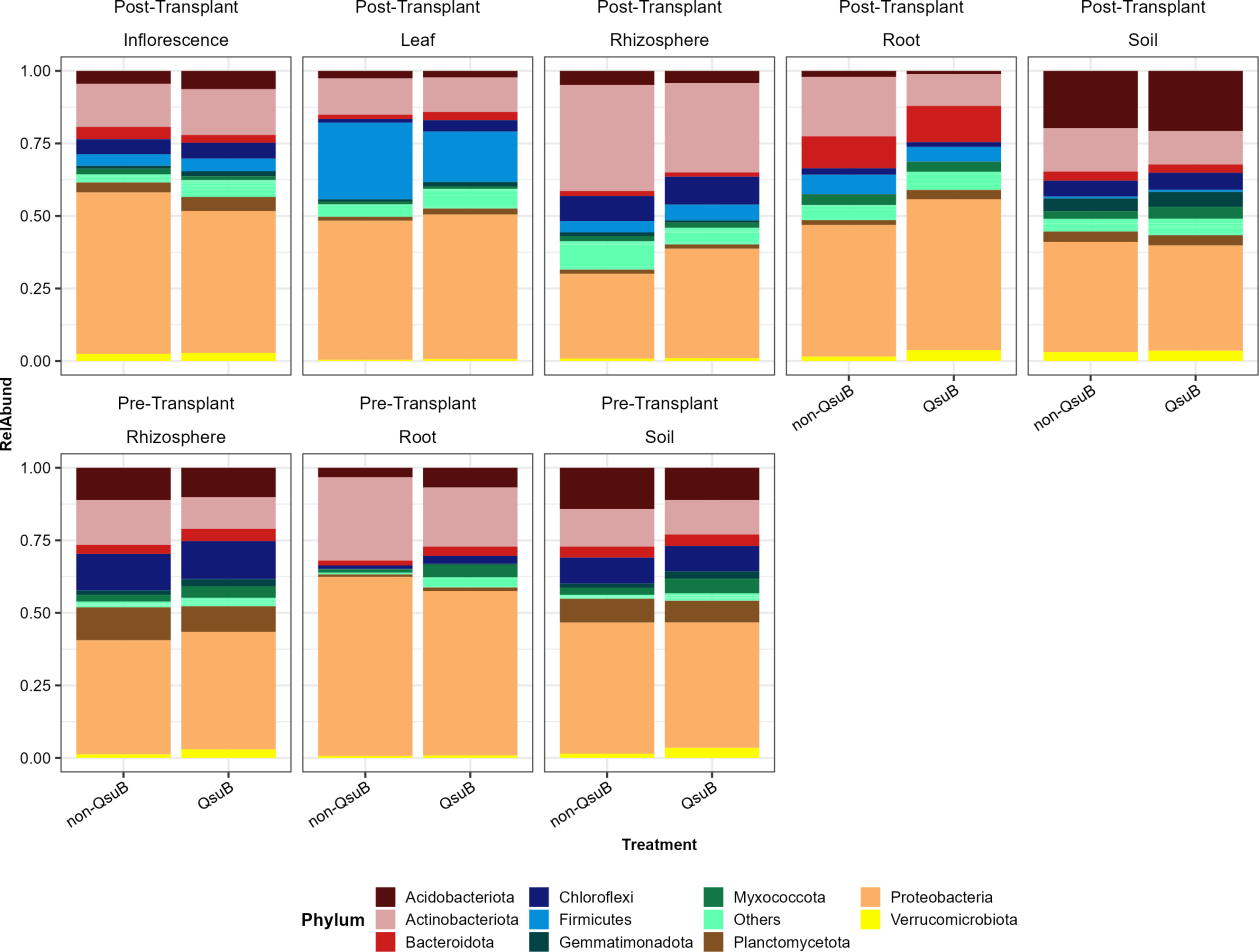
Lineage-level bacterial taxonomic distribution of samples from inflorescence, leaf, rhizosphere, root, and bulk soil niches of post-transplant and pre-transplant.

Overall, 47 bacterial lineages were detected in our samples, and the sum of top 10 most abundant lineages accounted for an average relative abundance of 95.64% among all samples. *Proteobacteria* and *Actinobacteria* were predominant bacterial lineages in our samples, with the average relative abundance of 45.47% and 17.53%, respectively (Fig. 5).

Bacterial communities of root and rhizosphere were significantly influenced by QsuB genotype. The only consistent trend associated with bacterial community was that QsuB plants had relatively fewer *Actinobacteria* compared to wild-type: 20.38% vs 28.72% (pre-transplant root), 10.93% vs 20.45% (post-transplant root), 10.90% vs 15.41% (pre-transplant rhizosphere), and 30.80% vs 36.56% (post-transplant rhizosphere) (Fig. 5). In pre-transplant rhizosphere and root samples, we observed a greater relative abundance of *Myxococcota* in QsuB than wild-type, but this trend was not shown in post-transplant samples. In post-transplant rhizosphere and root samples, we observed relatively more *Proteobacteria* in QsuB than wild-type, but this trend was not shown in pre-transplant samples (Fig. 5). These trends were not statistically significant.

The bacterial relative abundance of the top 10 orders is shown in Fig. S9. Some trends are numerically evident: compared to wild-type, QsuB plants had relatively more *Pseudonocardiales* in roots and more *Burkholderiales* in rhizosphere (Fig. S9). The greater alpha-diversity (richness and Shannon index) in QsuB plants than wild-type was likely explained by relatively more “Others” in pre-transplant root samples (28.90% in QsuB vs 14.02% in wild-type), in post-transplant inflorescence samples (45.27% in QsuB vs 37.30% in wild-type), and in post-transplant leaf sample (30.21% in QsuB vs 27.71% in wild-type) (Fig. S9).

### Indicator ASVs

We used differential abundance measurements between QsuB genotype and wild-type switchgrass to identify indicator ASVs. Consistent with our results, the Wilcoxon test commonly outputs a high number of significant ASVs compared to DESeq2 since the former is less stringent (43, 44). DESeq2 identified 14 and 15 bacterial biomarkers in pre- and post-transplant root samples, respectively, while Wilcoxon test identified 99 and 307 significant ASVs in those metadata subgroups. No indicator ASVs were detected in leaf fungal, while three (two in pre-transplant and one in post-transplant) were detected for rhizosphere bacterial samples by DESeq2, even though those samples’ microbial communities were significantly influenced by QsuB genotype (Fig. 3). Root sample “niche” was the only group that significantly influenced both fungal and bacterial communities (Fig. 3), so we focused on the fungal and bacterial indicator ASVs of root samples. Most indicator ASVs identified with DESeq2 were also identified with the Wilcoxon test.

**Fig 6:**
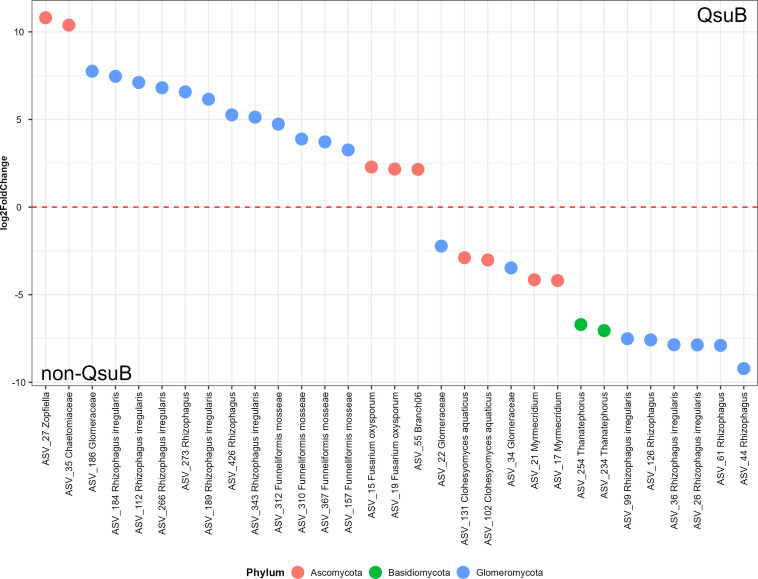
Differential expression analysis of fungal post-transplant root samples by DESeq2 displays the fungal amplicon sequence variants that are significantly differentially abundant between QsuB and non-QsuB wild-type plants. Different colors represent different phyla each sample belongs to. The ASVs above the dashed line (log_2_FoldChange > 0) are significantly more abundant in the QsuB traits, while the ASVs below the dashed line (log_2_FoldChange < 0) are significantly more abundant in the non-QsuB wild-type.

Thirty-one and thirty-two fungal indicator ASVs were identified in the pre- and post-transplant root samples, respectively. The majority of these (14 of 31 from pre-transplant and 20 of 32 from post-transplant) were *Glomeromycotina* (Fig. S10 and Fig. 6). All of the identified *Glomeromycotina* belonged to the arbuscular mycorrhizal fungi (AMF) family *Glomeraceae*. Four *Funneliformis* (AMF) indicator ASVs were identified in post-transplant roots but not in pre-transplant root samples.

Fifteen fungal indicator ASVs were detected both in pre- and post-transplant root samples. Six of them were associated with the QsuB genotype: ASV_27 (*Zopfiella*), ASV_35 (*Chaetomiaceae*), and ASV_112, 184, 189, 273 (*Rhizophagus*). Nine of them were associated with the wild-type switchgrass: ASV_21 (*Myrmecridium*), ASV_22, 34 (*Glomeraceae*), and ASV_26, 36, 44, 61, 99, 126 (*Rhizophagus*) (Fig. S10 and Fig. 6).

Fewer bacterial indicator ASVs were identified by DESeq2 compared to the fungal indicator ASVs. Bacterial indicator ASVs were dominated by *Proteobacteria* and *Actinobacteriota* in pre- and post-transplant root samples, respectively. In pre-transplant root samples, *Lentzea aerocolonigenes* (ASV_191 and 244) were associated with the QsuB plants, while *Amycolatopsis mediterranei* (ASV_46 and 69) were associated with non-QsuB wild-type plants (Fig. S11). Both species belong to *Pseudonocardiales*. In post-transplant root samples, *Streptomyces* (ASV_101) was the only *Actinobacteria* indicator associated with the QsuB plants (Fig. S12).

## DISCUSSION

In this research, we set out to assess whether switchgrass engineered for low lignin with the QsuB gene would impact the microbiome associated with different aboveground and belowground plant organs. As we hypothesized, our results indicate that QsuB-engineered plants impacted switchgrass-associated microbial community structure. Our results showed that the QsuB genotype influenced fungal and bacterial community structure. Specifically, QsuB plants had a significant impact on the fungal community in root and leaf samples and also a significant impact on belowground bacterial microbiomes in the root and rhizosphere. In contrast, we observed little impact of the QsuB genotype on inflorescence and bulk soil fungal or bacterial microbiomes.

### QsuB plants showed lower relative abundance and diversity of AMF

Arbuscular mycorrhizal fungi are obligate biotrophic plant mutualists that belong to *Glomeromycotina*. These fungi are known to be beneficial to plant nutrition and soil health by transporting nutrients (e.g., P and N) and water to plant hosts via their hyphal network, while stimulating and stabilizing soil organic matter (45–49). Under nutrient limitation, host plants are more dependent on AMF for nutrients (50, 51). In this study, we detected lower relative abundance of *Glomerales* (i.e., the most frequent AMF order detected in this study) sequences in the root samples from QsuB plants (both pre- and post-transplant).

The expression of QsuB in switchgrass results in the reduction of lignin and the accumulation of protocatechuate in biomass and improves biomass saccharification efficiency (16). Less lignin and more protocatechuate may have stimulated bacterial community activity and increased soil nutrient mineralization and turnover rates (52). More available nutrients may reduce the reliance of plants on AMF and may indirectly affect AMF colonization. For example, nutrient deficiency could trigger plant signaling, such as phenols, flavonoids, and sesquiterpenoids, and promote the growth of AMF appressorium (53). Five lower lignin transgenic lines of poplar with downregulated genes of monolignol biosynthesis pathway displayed a lower mycorrhizal colonization percentage than wild-type, and the authors proposed that the gene modifications in monolignol pathway impacted ectomycorrhizal colonization possibly by changing cell wall ultrastructure and decreasing the communication efficiency between plants and fungi (30).

### QsuB roots showed an increase in the relative abundance of *Sordariales* and *Hypocreales*

In this study, greater relative abundance of *Ascomycota* (e.g., *Sordariales* and *Hypocreales*) was detected in the root samples from QsuB plants compared to the wild-type. Previous work has identified *Sordariales* and *Hypocreales* as dominant decomposers in arable soil with long-term organic management practice (54), and we found these orders predominant in our samples. It is known that mycorrhizal and saprotrophic fungi compete for niche space and organic substrates (55–57). For example, Cao et al. (58) reported that AMF inhibited the population abundance and enzyme activity of saprotrophic fungi, possibly by reducing the availability of limiting nutrients. Increased accessibility to carbohydrates and soil nutrients may have stimulated *Sordariales* and *Hypocreales*, which were associated with the QsuB genotype.

Interestingly, in both root and leaf samples, QsuB plants had a higher relative abundance of *Hypocreales*. Six *Fusarium* (members of Hypocreales) ASVs were identified as indicator ASVs whose relative abundances significantly increased in the root and leaf samples of QsuB plants compared to the wild-type. This is of interest because many *Fusarium* species are known plant pathogens (59). In root samples, QsuB plants had relatively greater *Fusarium* in their rhizobiome compared to the wild-type: 9.81% vs 2.82% (pre-transplant) and 7.27% vs 1.97% (post-transplant); however, this trend was not obvious in the leaf samples (Fig. S13). The only two leaf *Fusarium* indicator ASVs were identified at the species level: *Fusarium oxysporum* (ASV_15 and 19), and they were also root *Fusarium* indicator ASVs. Soils are often the source of plant-associated *F. oxysporum* (60), and detached leaf assays showed that *F. oxysporum* might be benign or beneficial in switchgrass, even though other *Fusarium* species were pathogenic (61). In this study, *F. oxysporum* was the only *Fusarium* species identified in leaf samples associated with QsuB plants. Knowing the diversity of *Fusarium* species, the complexity of their function, and the limits of short amplicon sequencing, the actual roles of the *Fusarium* spp. in our study are difficult to discern (62).

In root samples, there were significantly more *Sordariales* in switchgrass QsuB plants than the wild-type, largely accounted for by *Zopfiella* (Fig. S13). Interestingly, some *Zopfiella* species have the potential to control plant disease by producing antifungal compounds and promote plant growth by increasing stress resistance (63, 64).

### QsuB plants hosted a greater richness and diversity of bacteria

We found that QsuB-engineered plants supported a significantly greater richness and diversity of bacteria in inflorescence, leaf, and root (pre-transplant) samples. Generally, bacteria are efficient at degrading simple substrates, while fungi are better equipped at decomposing recalcitrant organic matter, such as lignin (65). Lignin biodegradation starts with lignin depolymerization, which is predominantly performed by fungi (66). Therefore, compared to lignin, protocatechuate represents a more favorable growth substrate for bacteria to utilize, and bacteria are competitive for carbon and energy sources. Plants expressing QsuB accumulate inside tissues more protocatechuate that may stimulate the activity of the bacterial community. Diverse bacteria are indeed known to degrade protocatechuate including those in the order *Bacillales*, *Burkholderiales*, *Sphingomonadales*, and *Pseudomonadales* (67–70).

### Fewer *Actinobacteria* were detected in the root and rhizosphere samples of QsuB plants

In this study, the relative abundance of *Actinobacteria* detected in the root and rhizosphere samples of QsuB plants was lower than that of the wild-type. *Actinobacteria* are an important terrestrial group of detritus decomposers (65), which are able to degrade lignin materials (71). Given that QsuB-engineered switchgrass biosynthesizes less lignin, it may be expected to host a lower relative abundance of *Actinobacteria*. In post-transplant root samples, all non-QsuB-associated bacterial indicator ASVs showing significantly increased relative abundances were *Actinobacteria* (Fig. S9).

### The AMF and bacterial community dynamics may be interlinked

We have described how the QsuB genotype influenced the fungal communities, especially AMF, as well as the bacteria communities belowground. We posit that the change in the AMF community might be an important driver of the change observed in those bacterial communities. Previous studies have found that the AMF community composition was a significant contributor to determining the bacterial community composition (72), perhaps through changes in the root exudates composition and soil structure modification (65, 73). Interestingly, AMF-associated bacterial communities have been shown to be structured predominantly by AMF symbiont identity (*Glomus geosporum* or *Glomus constrictum*), rather than the host plant (*Plantago lanceolata* or *Hieracium pilosella*) (74). AMF hyphae release a variety of exudates, including carbohydrates, polyols, amino acids, amines, nucleic acids, organic acids, etc.; different AMF species or the same AMF under different abiotic conditions might have different metabolite profiles of hyphal exudates (75, 76). The carbon sources supplied by AMF have important roles in bacterial growth and distribution, so it is likely that AMF activity has an impact on the surrounding bacterial communities (76–78). AMF hyphae provide a scaffold bridging the soil and root microbiomes (79). The addition of protocatechuate to the culture medium inhibited primary root growth but increased lateral root numbers in Arabidopsis (80). The potential root morphology change may also influence AMF development and surrounding bacterial communities.

Some specific bacteria showed the same (positive or negative) response to AMF, even under different experimental setups. For example, in this study, root and rhizosphere samples from QsuB plants had relatively fewer AMF and *Actinobacteria*. This positive response of *Actinobacteria* to AMF has also been observed in other studies (76, 81). Recent work showed that QsuB switchgrass had no yield penalty compared to the wild-type under optimal irrigation in the field (82). However, as AMF and *Actinobacteria* are associated with plant drought resilience, this raises questions into how QsuB plants would fare under water-limiting conditions (83, 84).

### Importance of testing microbiome impacts in QsuB-engineered plants

Engineering the biofuel feedstock switchgrass with the QsuB gene is a promising strategy for reducing lignin content and improving saccharification. Our work highlights the importance of assessing QsuB genotype impact on the plant-associated microbiomes. We found that QsuB engineering changed plant physiology and its microbiomes, including some important functional microbial groups including AMF and *Actinobacteria*. Unfortunately, we did not obtain chemical data of the roots and surrounding rhizosphere, so the lignin and protocatechuate contents of specific belowground compartments are unknown. It is possible that the accumulation of protocatechuate altered plant cell osmolarity, and might further modify the root exudates, which may directly contribute to the microbial community dynamics. A longer-term field study of QsuB bioenergy plants with measurements of biomass yield, soil properties, and microbiome communities in different locations and climates could help confirm the promise and future of QsuB-engineered bioenergy crops under real-world agricultural scenarios.

### Conclusion

Less lignin content, more fermentable sugar yield, and greater biomass conversion efficiency to biofuels made QsuB genotype promising in wide application. As hypothesized, our study found that the QsuB-engineered plants impacted switchgrass-associated fungal and bacterial communities, especially those associated with the roots and rhizosphere. Importantly, the microbiome differences between QsuB plants and non-modified wild-type switchgrass did not appear to impact the relative abundances of putative switchgrass pathogens. However, the reduction in AMF diversity and relative abundance in QsuB plants are noteworthy and raise questions regarding how this could further impact plant performance under drought conditions and consequent soil physio-chemical properties. By characterizing the microbiome responses to QsuB genotype, we provide a baseline for evaluating QsuB and other bioengineered traits on plant-microbe interactions.

## SUPPLEMENTAL MATERIAL

Supplemental figures spectrum.01546-24-s0001.docx Fig. S1 to S13.

Supplemental tables spectrum.01546-24-s0002.docx Tables S1 and S2.

ASM does not own the copyrights to Supplemental Material that may be linked to, or accessed through, an article. The authors have granted ASM a non-exclusive, world-wide license to publish the Supplemental Material files. Please contact the corresponding author directly for reuse.
