# Supplementary material for: Genetic modification of the shikimate pathway to reduce lignin content in switchgrass (Panicum virgatum L.) significantly impacts plant microbiomes
Source: Microbiol Spectr. 2024 Nov 26;13(1):e01546-24. doi: 10.1128/spectrum.01546-24 (PMC11705929; doi:10.1128/spectrum.01546-24)
Supplement: Supplemental figures — Fig. S1 to S13. [file spectrum.01546-24-s0001.docx]

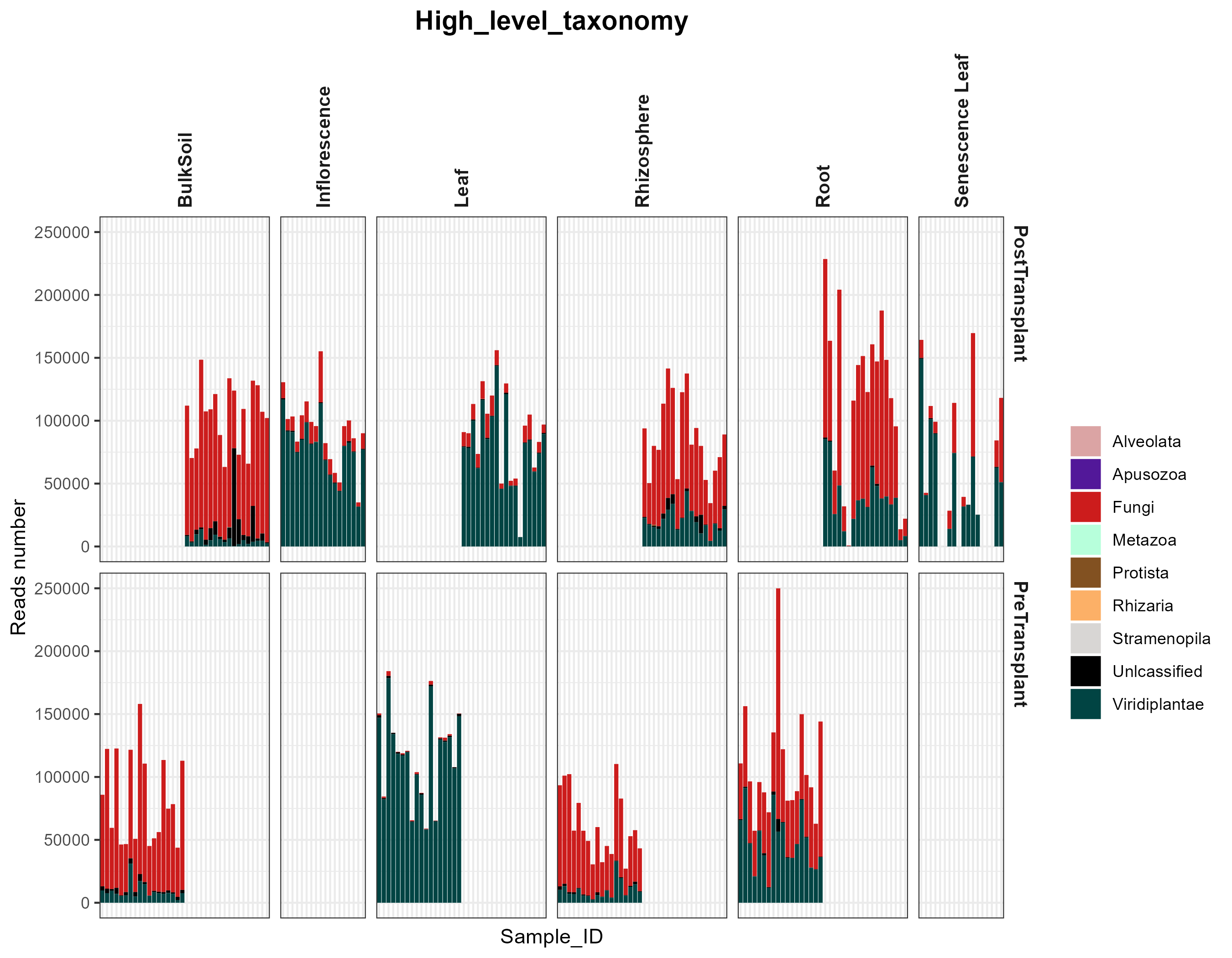


**Figure S1.** The total ITS rDNA sequence reads-number of each metadata group. Different colors illustrate different high-level-taxonomy.


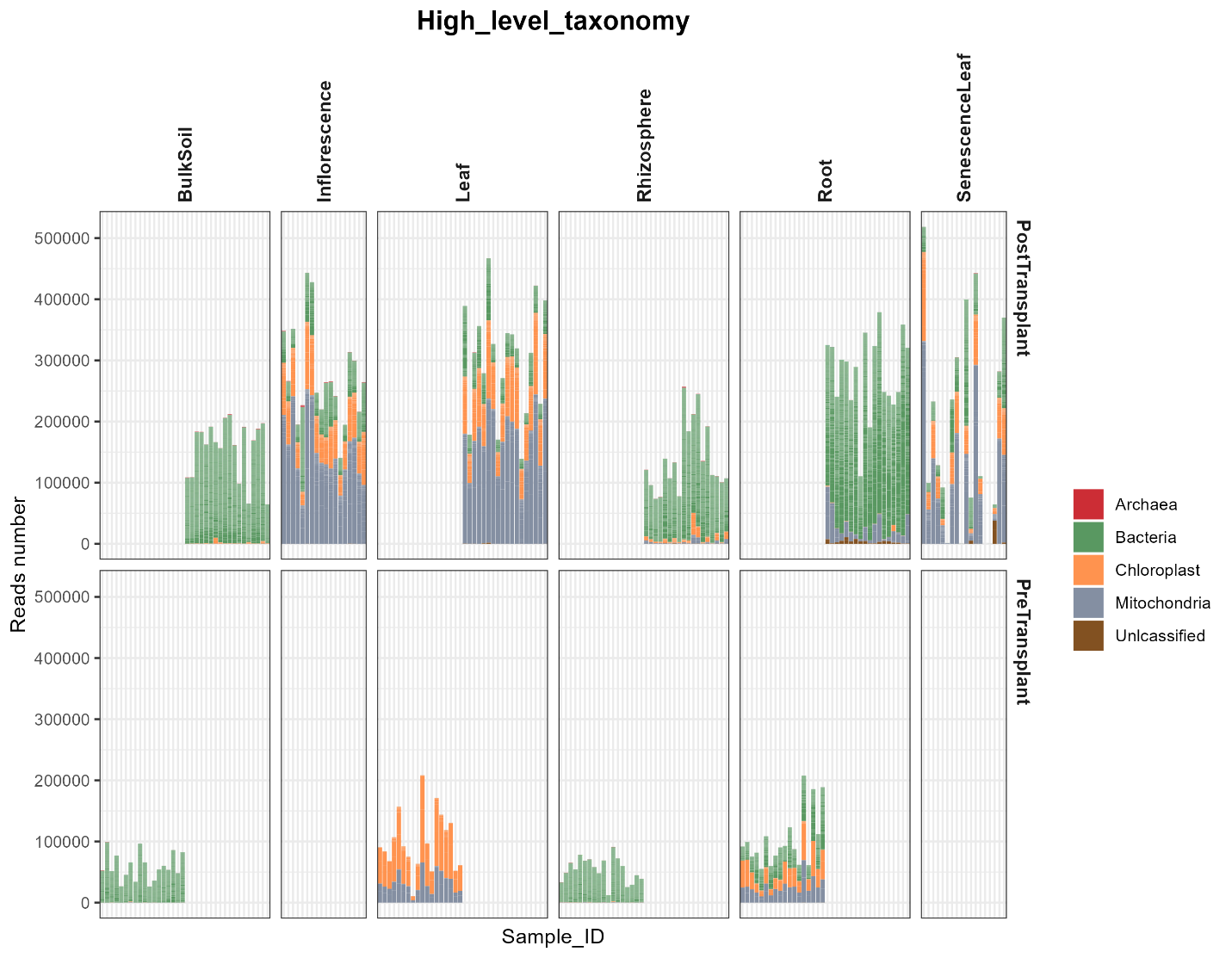


**Figure S2.** The total 16S rRNA sequence reads-number of each metadata group. Different colors illustrate different high-level-taxonomy.


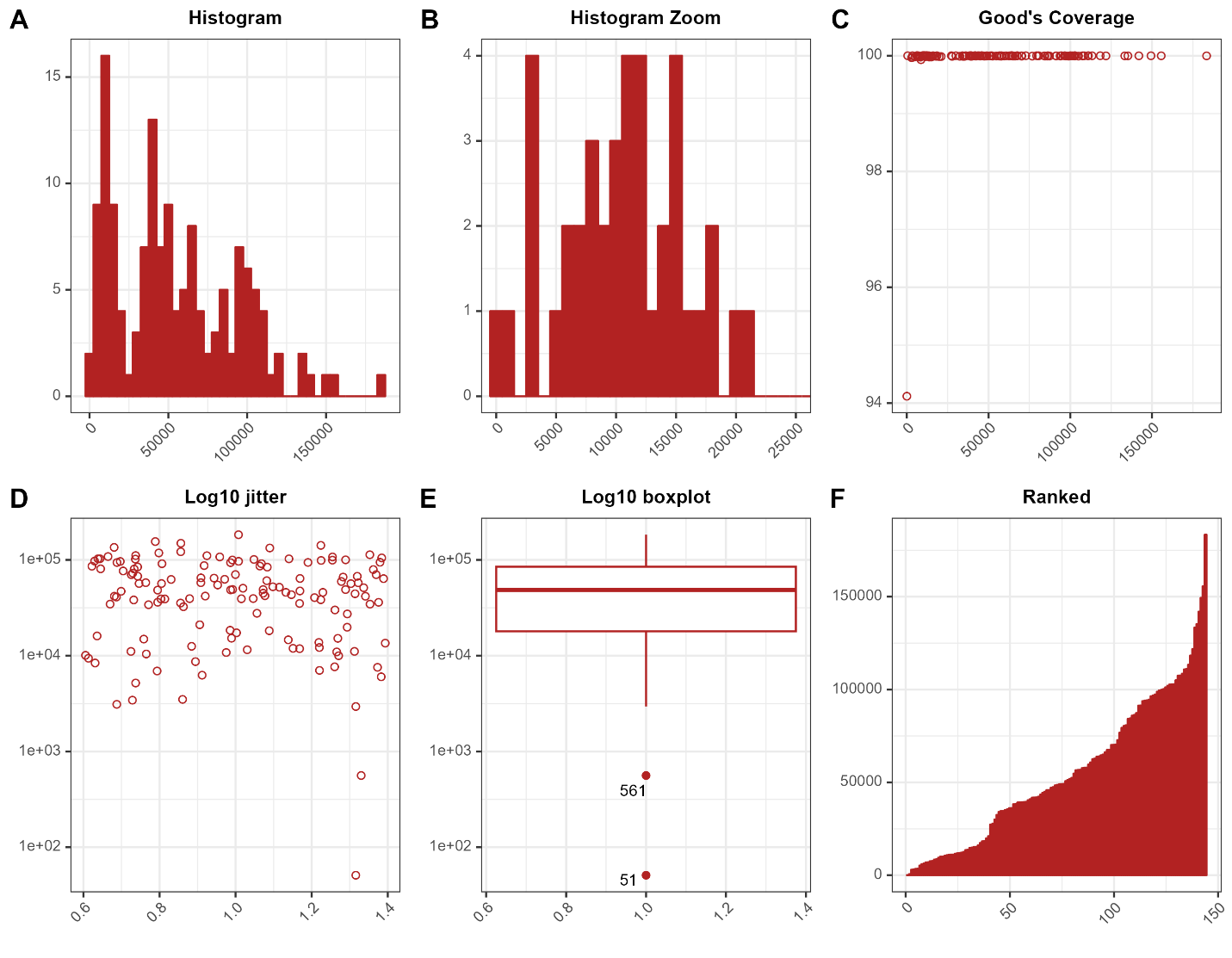


**Figure S3.** Graphic view of fungal rarefaction statistics with two outliers detected.


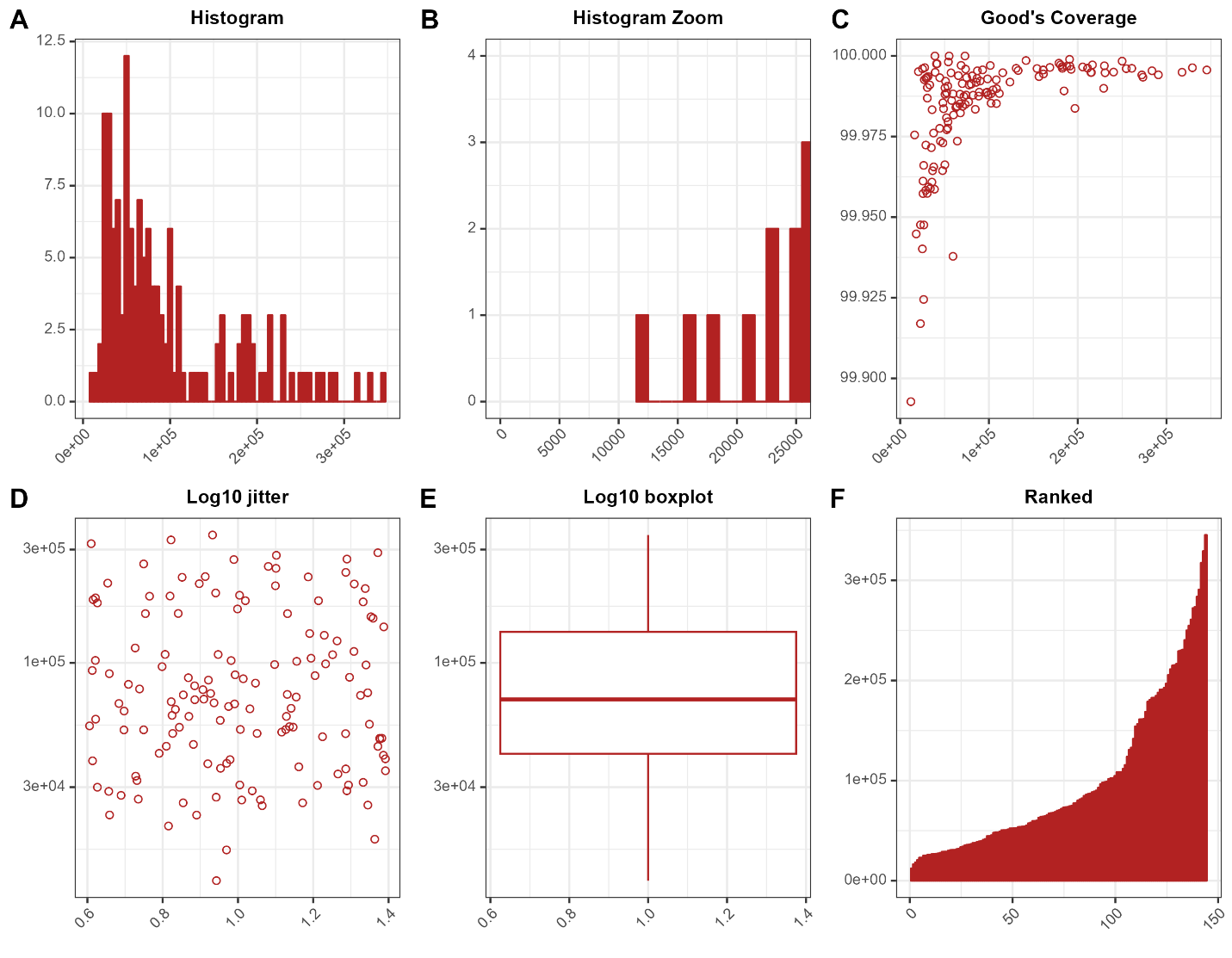


**Figure S4.** Graphic view of bacterial rarefaction statistics.


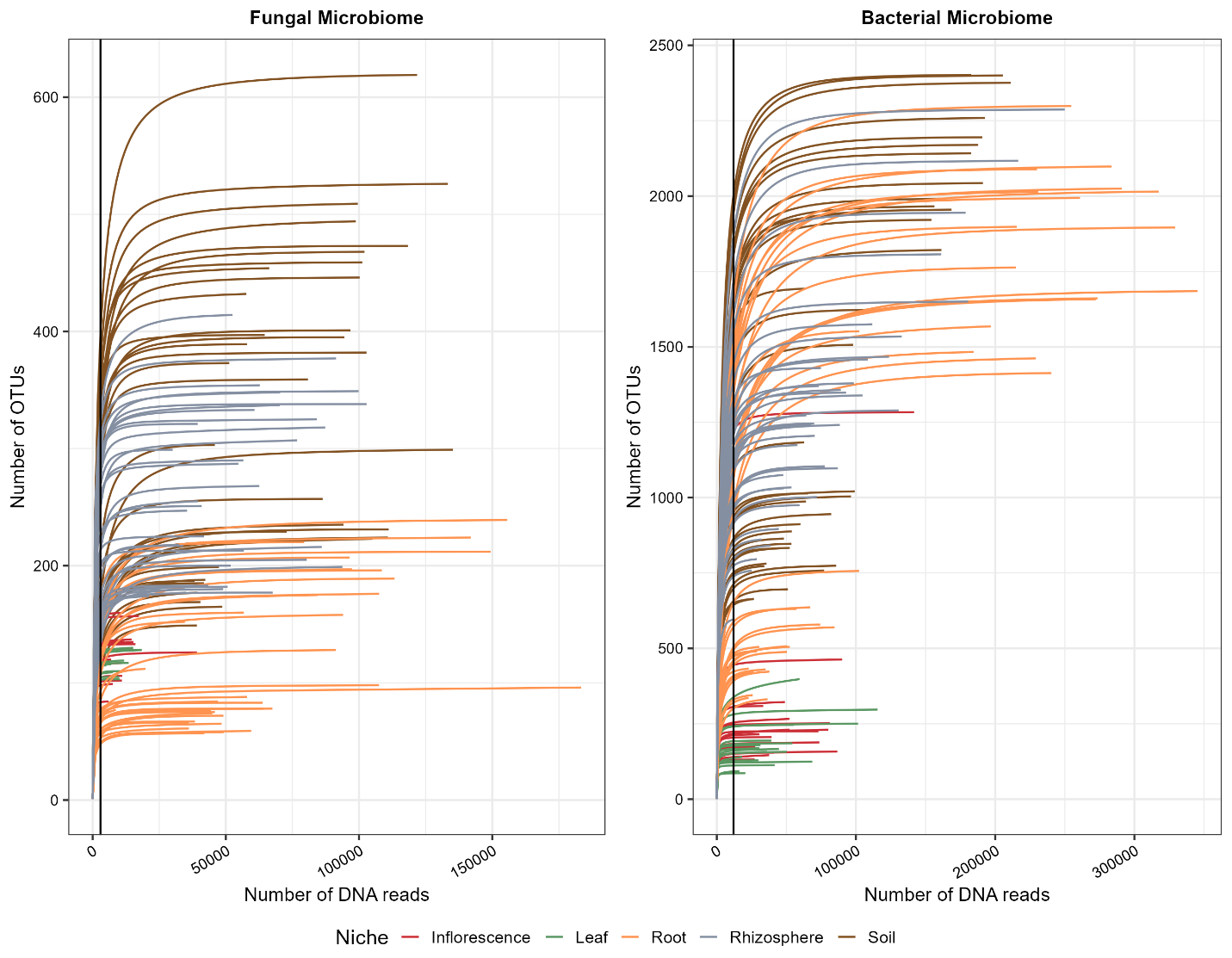


**Figure S5.** Rarefaction curves highlighting samples from different sampling niches for fungal and bacterial datasets.


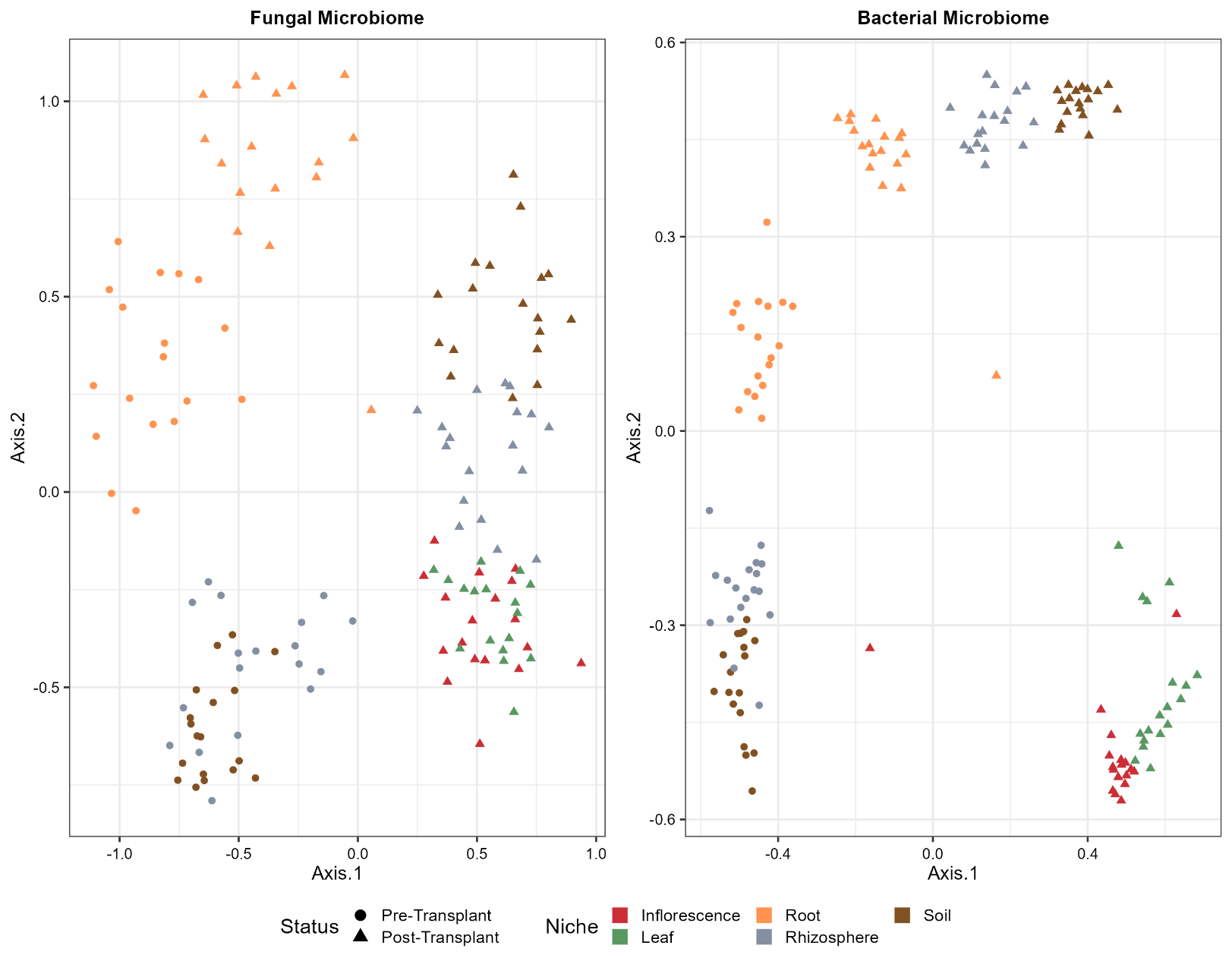


**Figure S6**. Nonmetric multidimensional scaling (NMDS) plots of fungal and bacterial microbiomes.


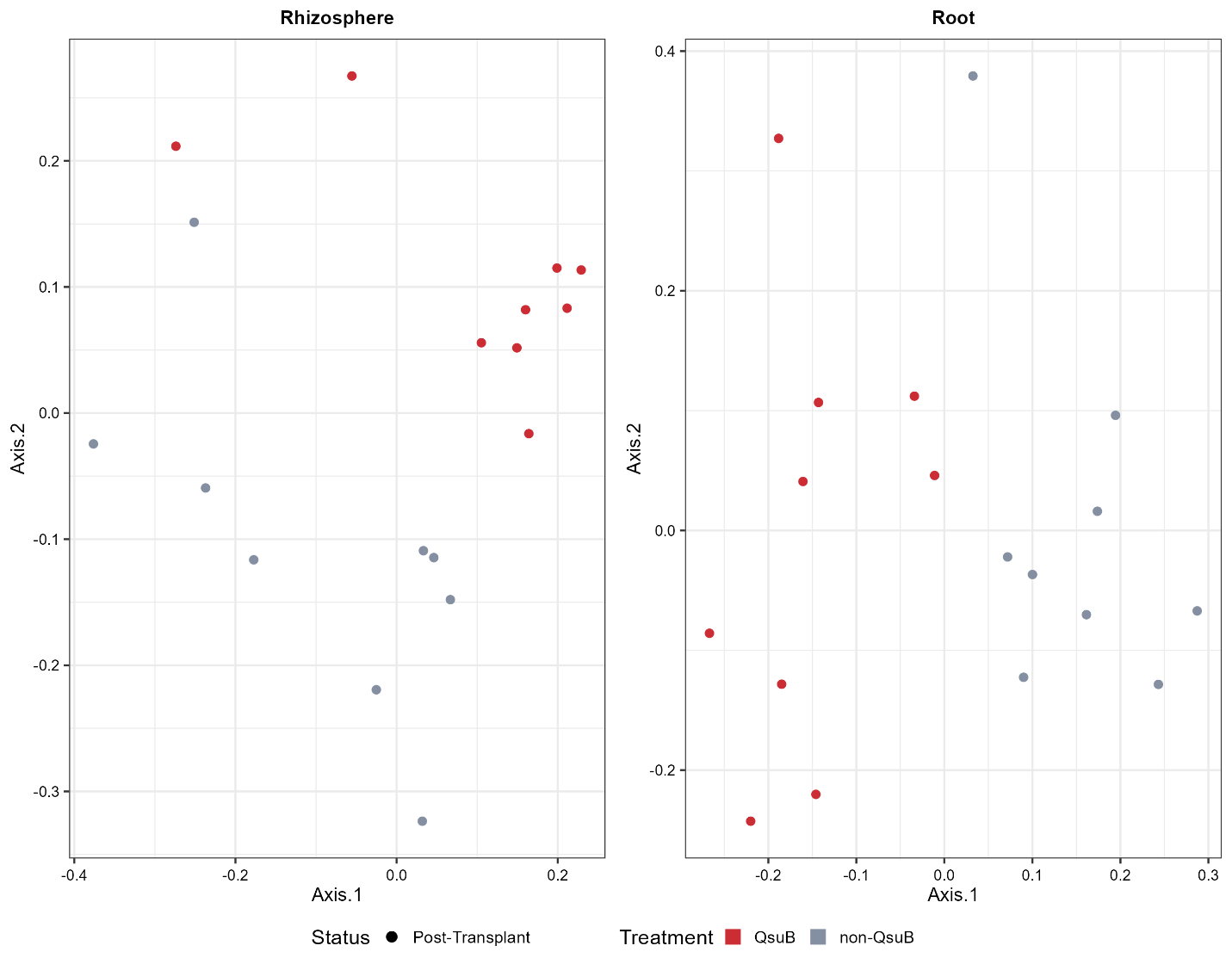


**Figure S7**. Principal coordinate analysis (PCoA) ordinations of bacterial communities sampled from rhizosphere and root niches at Post-Transplant.


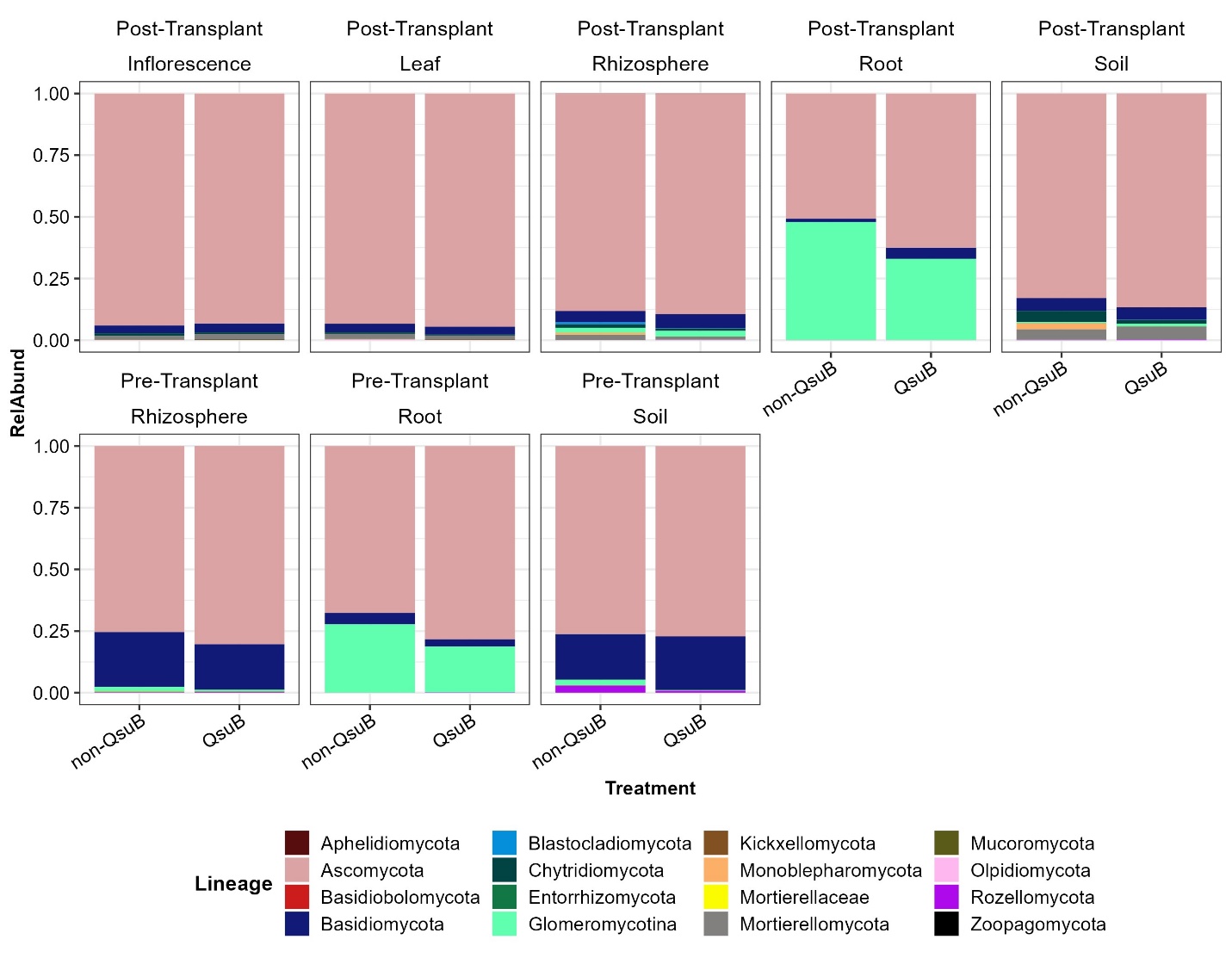


**Figure S8**. Lineage-level fungal taxonomic distribution of samples from inflorescence, leaf, rhizosphere, root, and bulk soil niches of Post-Transplant and Pre-Transplant.


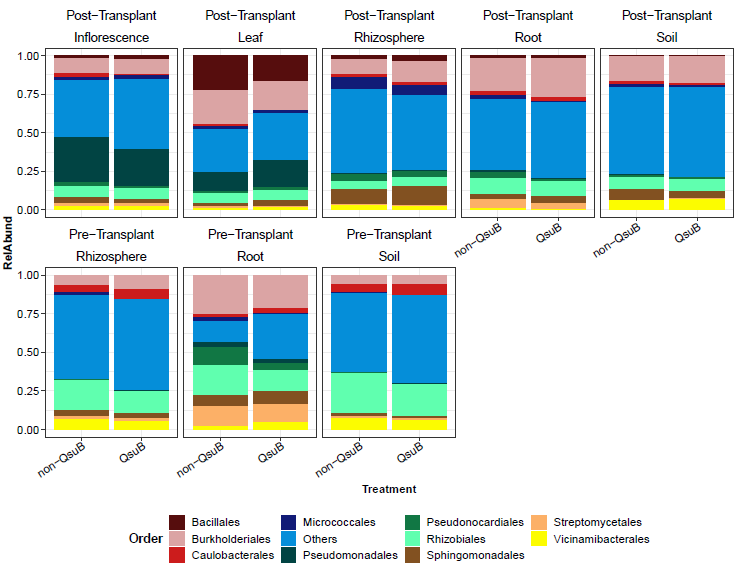


**Figure S9**. Order-level bacterial taxonomic distribution of samples from inflorescence, leaf, rhizosphere, root, and bulk soil niches of Post-Transplant and Pre-Transplant.


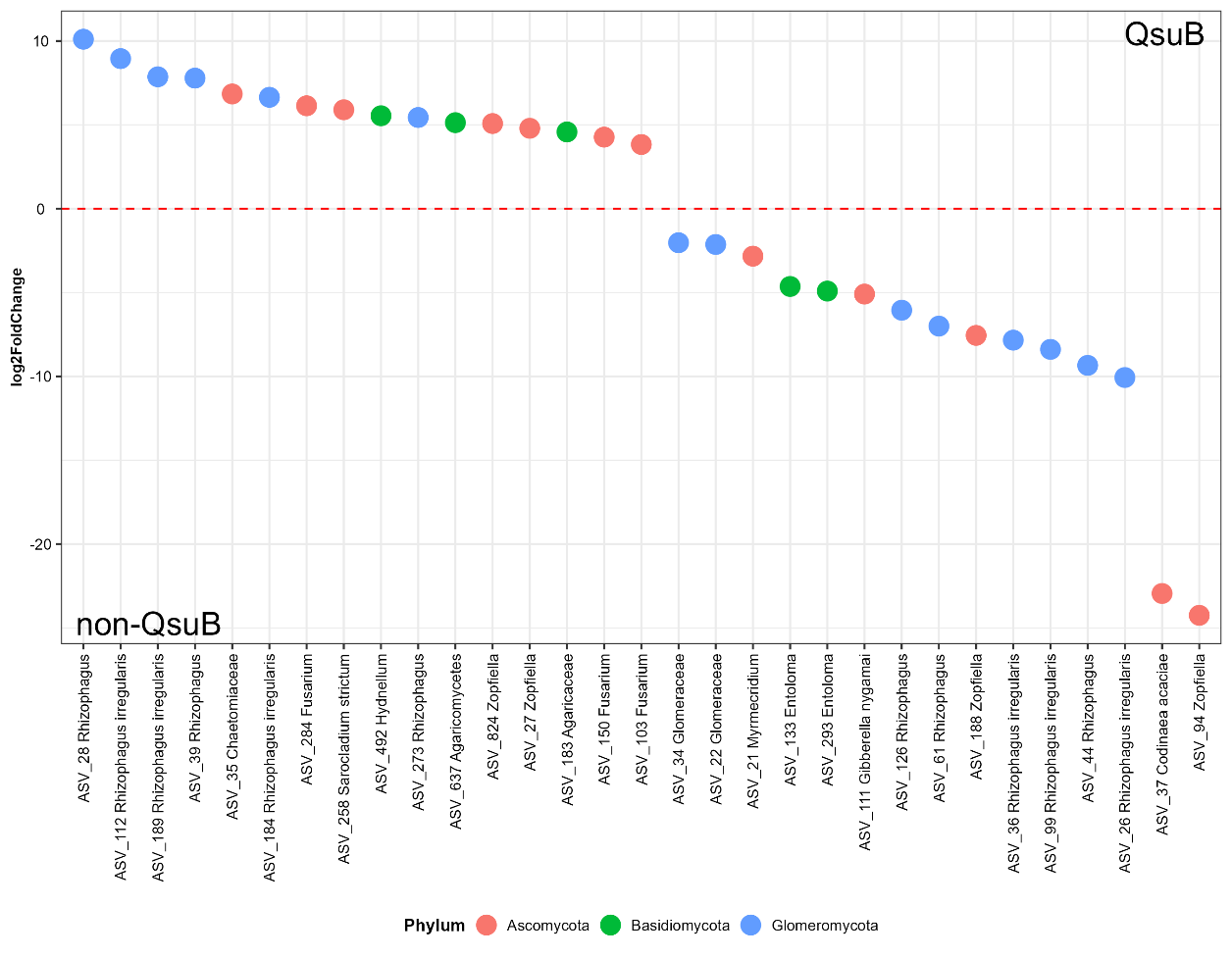


**Figure S10**. Differential analysis of fungal Pre-Transplant root samples by DESeq2 displays the fungal amplicon sequence variants (ASVs) that are significantly differentially abundant between QsuB and non-QsuB wildtype plants. Different colors represent different phyla each sample belongs to. The ASVs above the dash line (log_2_FoldChange > 0) are significantly more abundant in the QsuB traits, while the ASVs below the dash line (log_2_FoldChange < 0) are significantly more abundant in the non-QsuB wildtype.


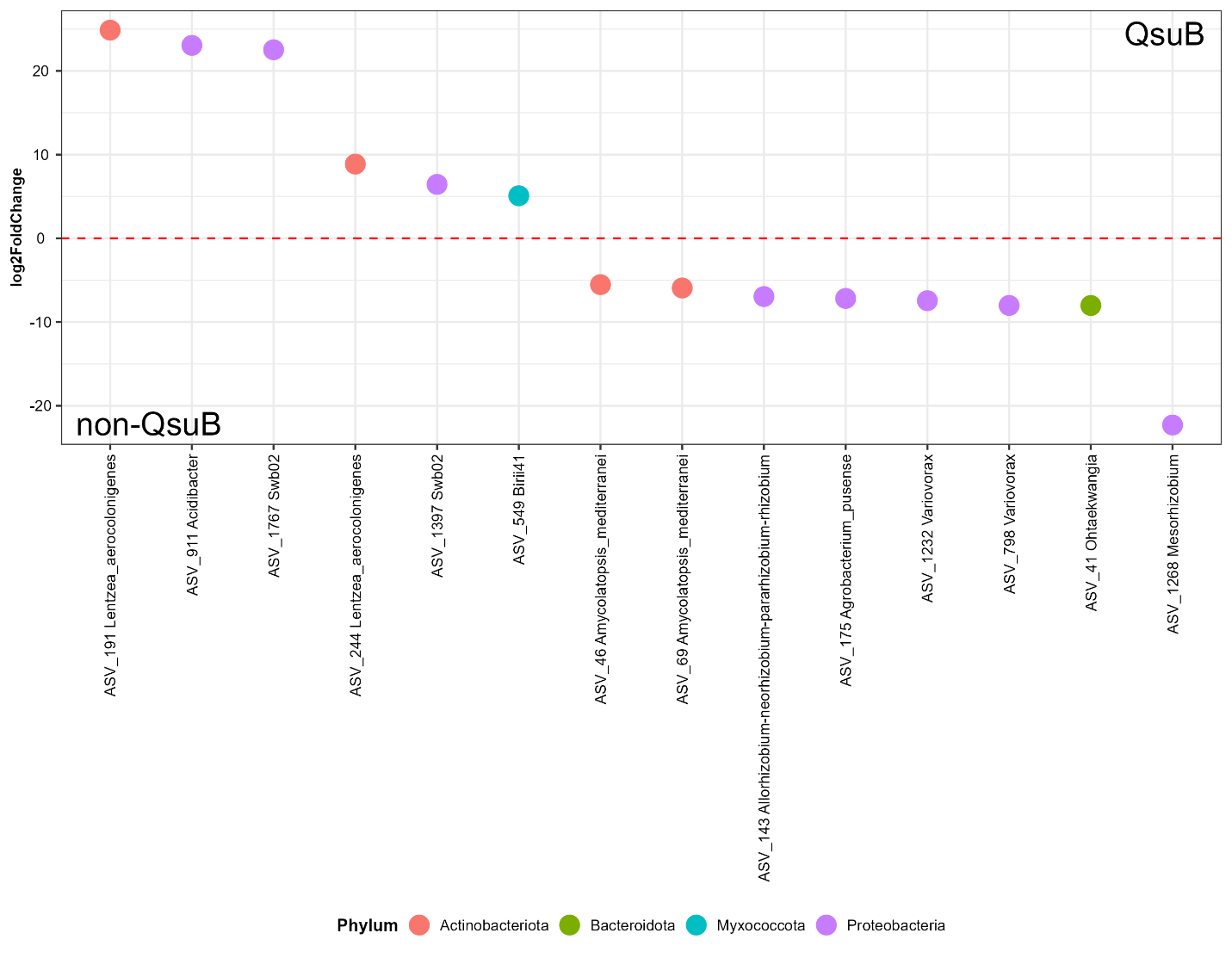


**Figure S11**. Differential analysis of bacterial Pre-Transplant root samples by DESeq2 displays the bacterial amplicon sequence variants (ASVs) that are significantly differentially abundant between QsuB and non-QsuB wildtype plants. Different colors represent different phyla each sample belongs to. The ASVs above the dash line (log_2_FoldChange > 0) are significantly more abundant in the QsuB traits, while the ASVs below the dash line (log_2_FoldChange < 0) are significantly more abundant in the non-QsuB wild type.


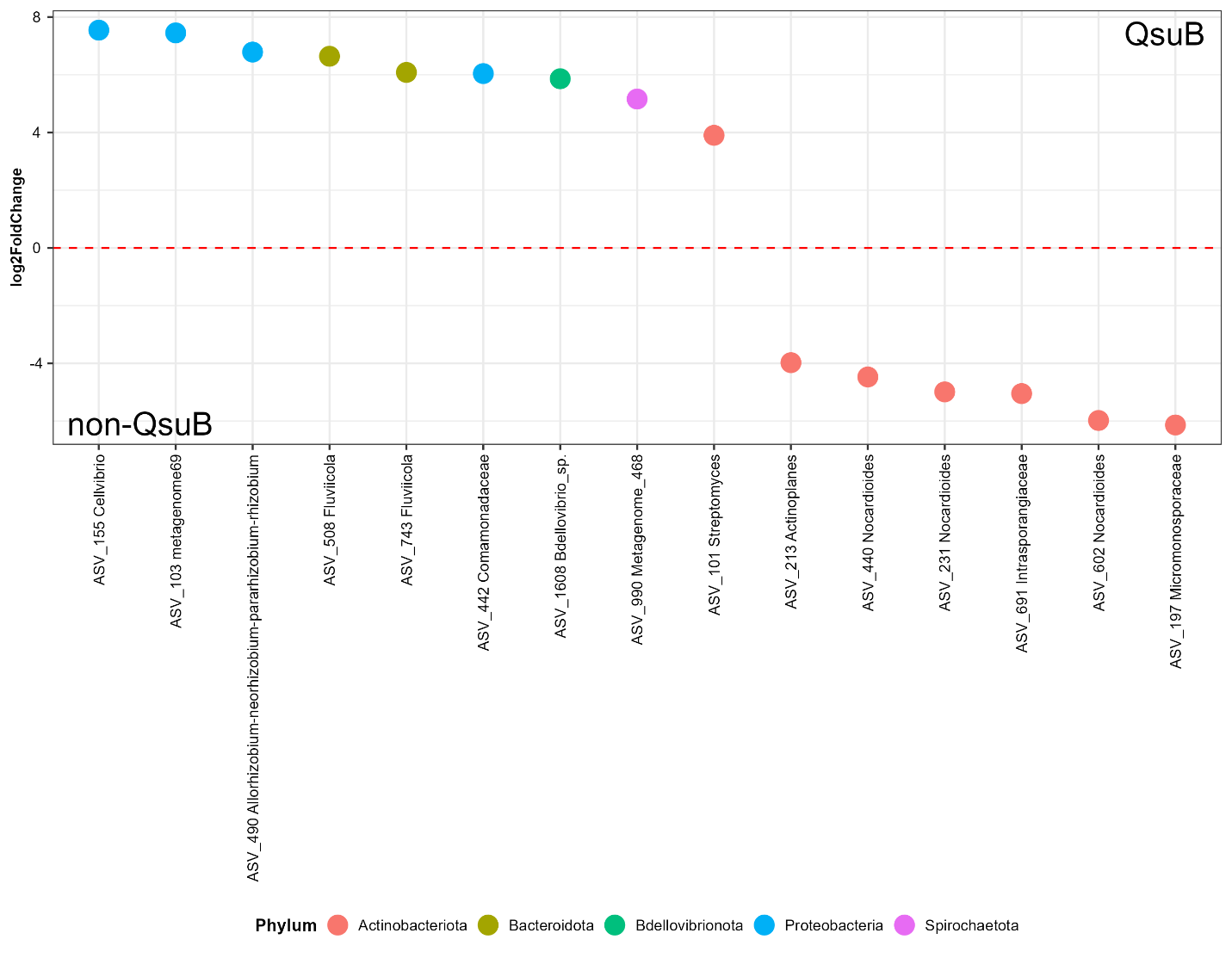


**Figure S12**. Differential analysis of bacterial Post-Transplant root samples by DESeq2 displays the bacterial amplicon sequence variants (ASVs) that are significantly differentially abundant between QsuB and non-QsuB wildtype plants. Different colors represent different phyla each sample belongs to. The ASVs above the dash line (log_2_FoldChange > 0) are significantly more abundant in the QsuB traits, while the ASVs below the dash line (log_2_FoldChange < 0) are significantly more abundant in the non-QsuB wildtype.


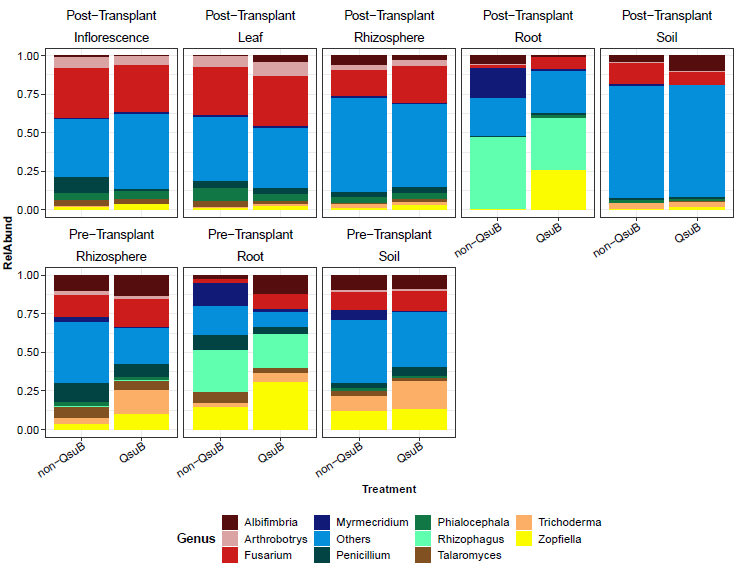


**Figure S13**. Genus-level fungal taxonomic distribution of samples from inflorescence, leaf, rhizosphere, root, and bulk soil niches
