# Supplementary material for: Genetic modification of the shikimate pathway to reduce lignin content in switchgrass (Panicum virgatum L.) significantly impacts plant microbiomes
Source: Microbiol Spectr. 2024 Nov 26;13(1):e01546-24. doi: 10.1128/spectrum.01546-24 (PMC11705929; doi:10.1128/spectrum.01546-24)
Supplement: Supplemental tables — Tables S1 and S2. [file spectrum.01546-24-s0002.docx]

**Table S1**. PERMANOVA (permutational multivariate analysis of variance) tests for fungal and bacterial beta-diversity differences using Bray-Curtis distance ordinations.

| Samples | Source | R^2^ | *p.* adj |
| --- | --- | --- | --- |
| Fungi  in  Roots | Treatment | 0.2309 | *0.0015* |
|  | Status | 0.1768 | *0.0015* |
|  | Treatment × Status | 0.0647 | *0.0030* |
|  | Residual | 0.5276 |  |
| Fungi in  post-transplant Leaf | Treatment | 0.0751 | *0.041* |
|  | Residual | 0.9249 |  |
| Bacteria  in  Root | Treatment | 0.0567 | *0.0030* |
|  | Status | 0.2056 | *0.0030* |
|  | Treatment × Status | 0.0435 | *0.0190* |
|  | Residual | 0.6942 |  |
| Bacteria  in  Rhizosphere | Treatment | 0.0415 | *0.0290* |
|  | Status | 0.2131 | *0.0030* |
|  | Treatment × Status | 0.0383 | *0.0290* |
|  | Residual | 0.7071 |  |
| Bacteria in  post-transplant Root | Treatment | 0.1117 | *0.0010* |
|  | Residual | 0.8883 |  |
| Bacteria in  post-transplant Rhizosphere | Treatment  Residual | 0.0856  0.9144 | *0.0030* |
|  |  |  |  |
|  |  |  |  |

**Table S2**. Permutational multivariate analysis of variance (betadisper) tests for fungal and bacterial beta-diversity differences using Bray-Curtis distance ordinations.

| Samples | Source | F | *p* |
| --- | --- | --- | --- |
| Fungi in  Roots | Treatment | 15.937 | *0.0006* |
|  | Status | 0.2383 | *0.6307* |
| Fungi in  post-transplant Leaf | Treatment | 2.1346 | *0.1694* |
|  |  |  |  |
| Bacteria in  Root | Treatment | 3.2075 | *0.0844* |
|  | Status | 7.7488 | *0.0087* |
| Bacteria in  Rhizosphere | Treatment  Status | 3.3870  10.545 | *0.0745*  *0.0021* |
| Bacteria in  post-transplant Root | Treatment | 0.1188 | *0.749* |
| Bacteria in  post-transplant Rhizosphere | Treatment | 0.437 | *0.518* |
|  |  |  |  |
